# Biofortification of Sea Bream Fillets with Artichoke Polyphenols: Effects on Antioxidant Capacity, Shelf Life, and Sensory Quality

**DOI:** 10.3390/foods15010175

**Published:** 2026-01-05

**Authors:** Rossella Vadalà, Giovanna Lo Vecchio, Laura De Maria, Daniela Metro, Roberta Tardugno, Nicola Cicero, Rosaria Costa

**Affiliations:** 1 Department of Biomedical, Dental, Morphological, and Functional Image Sciences (BIOMORF), University of Messina, Via Consolare Valeria 1, 98125 Messina, Italy; rvadala@unime.it (R.V.); giovanna.lovecchio@unime.it (G.L.V.); laura.demaria@unime.it (L.D.M.); daniela.metro@unime.it (D.M.); costar@unime.it (R.C.); 2 Department of Pharmacy-Drug Sciences, University of Bari ‘Aldo Moro’, Via Orabona 4, 70125 Bari, Italy; roberta.tardugno@uniba.it; 3 Institute for Agriculture and Forestry Systems in the Mediterranean, National Research Council of Italy, Via Empedocle 58, 95128 Catania, Italy; 4 Science4life srl, University of Messina, 98168 Messina, Italy

**Keywords:** artichoke, biofortification, waste recycling, sensory analysis, shelf life, functional food, vacuum immersion, electroporation, sea bream

## Abstract

A functional seafood product was obtained by biofortifying fish fillets with polyphenols extracted from artichoke by-products. Two fortification techniques—vacuum immersion (VI) and spray coating followed by electroporation (SCE)—were applied and compared with untreated control (CTR) samples. The treated by vacuum immersion (TRT-VI) group showed the highest antioxidant power (DPPH scavenging: 42.5 ± 3.2% vs. 19.6 ± 1.5% in CTR. Colorimetry revealed significant shifts in lightness (L*), red-green component (a*), and yellow-green component (b*) values in raw and cooked fillets. In the TRT-VI group the microbiological shelf life was extended by approximately 4–5 days. Sensory analysis revealed that, despite of bitterness and astringency, key attributes were maintained. Phenolic profiling identified caffeoylquinic acids as the dominant compounds in both artichoke extracts and fortified fillets (range 0.5–304.5 mg·100 g^−1^). In this study the development of functional seafood products has been implemented through the valorisation of an agri-food by-product and the exploitation of emerging fortification technologies. Key outputs include the assessment of the nutritional value of the fortified fish fillets and the extension of shelf life without compromising key sensory attributes. Future studies could be directed toward the optimisation of formulations and bioavailability of the incorporated polyphenols.

## 1. Introduction

Over the last two decades, functional foods have emerged as a central focus of research and innovation within the broader fields of nutritional science and preventive medicine [1,2]. Beyond providing essential nutrients, these products are specifically formulated to address the nutritional requirements of targeted consumer groups, thereby contributing to health promotion, disease risk reduction, and overall well-being. The growing body of scientific evidence supporting functional foods, together with their increasing commercial relevance, reflects a paradigmatic shift in consumer behaviour toward health-oriented dietary patterns [3].

In parallel, the global functional food market has experienced substantial growth, reaching approximately 347 billion USD in 2024, with further expansion projected over the next decade [4,5]. Within this rapidly evolving sector, fish- and seafood-based functional foods represent a particularly dynamic and expanding segment of production. In 2024, the global functional seafood market, a category that includes fish products enriched with health-promoting ingredients, was valued at about 15.6 billion USD and is expected to grow steadily through 2034 (projected to reach ca. 25.8 billion USD), driven by increasing consumer demand for omega-3-rich and nutraceutical products [5].

This trend is mirrored in European seafood markets, where total consumer spending on fishery and aquaculture products reached approximately 62.8 billion EUR in 2024, reflecting sustained demand for value-added and high-quality seafood across the region. Within Europe, fish and seafood are increasingly perceived not only as sources of essential nutrients but also as platforms for functional enrichment, a shift that supports the development of fortified seafood products tailored to health-oriented consumers. Among commercially relevant species, farmed gilthead sea bream (*Sparus aurata*) represents a strategic candidate for functional seafood development due to its widespread production in Mediterranean aquaculture, year-round availability, and strong consumer acceptance in European markets [6]. The use of aquaculture-derived sea bream ensures reduced biological variability compared to wild-caught fish, thereby improving the reproducibility and scalability of biofortification strategies at an industrial level [6]. Moreover, sea bream fillets exhibit favourable technological characteristics, including compact muscle structure and good processing yield, which support the application of post-harvest functionalization techniques aimed at enhancing nutritional and shelf-life properties [7,8].

Biofortification consists of the enrichment of foods with naturally derived bioactive compounds such as vitamins (D, B12), minerals (iodine, selenium), long-chain n-3 PUFAs, and polyphenolic phytochemicals [7,8,9]. In the fishery and seafood sector, biofortification strategies have gained increasing relevance not only for nutritional enhancement but also for improving oxidative stability and extending shelf life, which are critical factors for the industrial viability of functional fish products [10]. As a result, the production of biofortified seafood is progressively transitioning from laboratory-scale investigations to pilot and pre-industrial applications, reflecting the growing maturity of this sector within the functional food market [10].

From a nutritional and preventive medicine perspective, functional seafood products have attracted particular interest in relation to neuroprotection and cognitive health [11]. Epidemiological and experimental evidence indicates that neurodegenerative processes are closely associated with chronic oxidative stress, neuroinflammation, and neuronal apoptosis, all of which can be significantly modulated by dietary components [11]. Marine-derived n-3 PUFAs, especially EPA and DHA, have demonstrated neuroprotective effects through the reduction in reactive oxygen species and the modulation of intracellular signalling pathways involved in cell survival and antioxidant defence [12,13].

In this context, plant-derived polyphenols have emerged as complementary bioactives capable of enhancing the functional potential of fish-based products. *Cynara scolymus* L. (artichoke) has been extensively characterised as a rich source of phenolic compounds, including luteolin and caffeoylquinic acids, with well-documented antioxidant and anti-inflammatory properties [14]. Importantly, growing evidence suggests that the combined administration of omega-3 fatty acids and polyphenols may result in synergistic effects, leading to enhanced modulation of oxidative stress, inflammatory responses, and pro-survival signalling pathways such as PI3K/AKT [15,16,17].

This synergistic approach has stimulated an increasing interest in the co-formulation of marine and botanical bioactive molecules in functional seafood products. Recent studies have demonstrated the technical feasibility of fortifying fish matrices with plant-derived extracts, reporting positive outcomes in terms of nutritional quality, oxidative stability, and sensory acceptability [18,19,20]. These findings support the development of next-generation functional seafood products that integrate sustainability, industrial feasibility, and targeted health benefits.

In this scenario, the main goal of the present work was to deliver a functional food with enhanced nutraceutical properties (increased phenolic content, prolonged shelf life, enhanced sensory quality) while maintaining an acceptable level of palatability.

The specific aims of the work were as follows: (i) to develop an effective biofortification strategy to be applied to farmed sea bream; (ii) to use antioxidants recovered from artichoke scraps to enrich fish fillets. To fulfil these tasks, sustainable extraction methodologies, i.e., enzymatic and ultrasound, were applied. Furthermore, two distinct fortification techniques were implemented, namely vacuum immersion (VI) and spray coating followed by electroporation (SCE). These methodologies were selected to maximise polyphenol content and post-cooking retention, thereby improving antioxidant capacity, shelf-life stability, and sensory acceptability of the final product. Ultimately, this research seeks to deliver a functional seafood prototype that integrates marine and botanical bioactive compounds into a consumer-accepted product for health-focused nutrition. This approach was designed to enhance the nutritional profile of fish fillets while targeting the specific dietary requirements of individuals affected by neurodegenerative disease.

## 2. Materials and Methods

### 2.1. Fish Samples

A total of 60 fillet samples of farmed sea bream (*Sparus aurata*) were obtained from the VRM Group (Verona, Italy). The samples belonged to Lot M1, originating from a recirculating aquaculture system (RAS) production unit of VRM establishment code IT 027VR-123; tanks V 1–24). Fish were filleted immediately after harvest and weighed (on average 120 g each) under standardised industrial conditions to ensure product homogeneity. Each batch was labelled with a lot number and freezing date to guarantee full traceability. The samples were delivered to the laboratories of the University of Messina (Italy) in August 2025, under strict cold-chain conditions to preserve their integrity during transport. Upon arrival, all fillets were stored in a dedicated −20 °C freezer until use. Storage conditions were continuously monitored to prevent temperature fluctuations and ensure product stability over time. Prior to the experimental activities, the samples were thawed at a controlled temperature of +20 °C to minimise structural alterations and ensure reproducibility across treatments. This standardised handling ensured consistent quality and reliability of the samples for subsequent analytical and experimental assays.

### 2.2. Artichoke Waste

Artichoke by-products consisted of leaves, stems, and external bracts and were procured at local fruit markets. The material was pretreated as follows: it was preliminarily oven-dried at 90 °C for 3 h and subsequently at 60 °C for 7 h, a drying protocol designed to ensure effective moisture removal while limiting thermal degradation of bioactive compounds. At the end of the drying process, the material was fully dried, reaching a residual moisture content below 8% (*w*/*w*). Moisture content was determined using the gravimetric oven-drying method, by drying samples to constant weight at 105 °C. This moisture level is considered suitable for milling and for use as input material in extraction processes. The dried material was then ground into powder using a knife mill (Retsch Grindomix GM 200, Haan, Germany) and passed through an optical granulometer (Haver Computerized Particle Analysis CPA 2-1, Haver and Boecker OHG, Oelde, Germany). The average particle size was assessed as 1.4 mm in diameter. Thereafter, the obtained powder underwent ultrasound-assisted enzymatic extraction: the powder was suspended in water (1:10, *w*/*v*) and sonicated in an ultrasonic bath (model LBS1, Falc Instruments S.r.l., Treviglio (BG), Italy) for 20 min at 20 kHz and 0.4 W mL^−1^. The sonicated solution was adjusted to pH 5 and supplemented with enzymes, namely pectinase and cellulase (pectinase = 3 mg/20 mg d.w.; cellulase = 35 mg/20 g d.w.), and incubated for 8 h at 40 °C. These enzymes were selected for their ability to hydrolyse the structural components of artichoke tissues, particularly cellulose in the plant cell wall and pectin in the middle lamella, thereby promoting the release of bound phenolic compounds. The obtained extract, hereafter referred to as AE, was then ready for the fortification process.

### 2.3. Fortification of Fish Fillets

Two distinct procedures were used for the fortification of fish fillets: (i) Vacuum Immersion (VI)—fish fillets were immersed in the AE and brought to a vacuum condition (−70 KPa for 20 min) after a period of 15 min of immersion at atmospheric pressure. During the vacuum stage, the air inside the fish tissue was removed. Upon re-pressurisation the artichoke extract penetrates more thoroughly into the fish tissue; (ii) Spray Coating combined with Electroporation (SCE)—the fillet is placed on a sanitised tray, and it is sprayed with the AE solution. About 5–10 mL of extract is sprayed on both sides of the fish fillet. The latter is left to stand for 1–2 min ca. The electroporation was carried out using a lab-scale fabricated prototype device. The system consisted of a custom-built chamber in polycarbonate, equipped with parallel stainless-steel plate electrodes (2 cm gap) connected to a high-voltage pulse generator. The operating conditions were set as follows: electric field strength of 2.0 kV/cm, pulse duration of 50 μs, with a number of 20 pulses, and temperature was maintained at 20 °C. Finally, the fillets were allowed to stand at 4 °C for 30 min. A control (CTR) group did not undergo fortification and was kept neat. So, all fish samples can be categorised as follows:

- TRT-VI (treated by vacuum immersion), *n* = 20
- TRT-SCE (treated by spray coating followed by electroporation), *n* = 20
- CTR, *n* = 20

### 2.4. DPPH Scavenging Activity

The antioxidant activity of the samples was evaluated using the 2,2-diphenyl-1-picrylhydrazyl (DPPH) radical scavenging assay. Aliquots (0.1 mL) of methanolic extracts obtained from raw fish fillet samples belonging to the three experimental groups (CTR, TRT-VI, and TRT-SCE) were mixed with 3.9 mL of DPPH solution. Each mixture was incubated for 30 min in the dark at room temperature, and absorbance was measured spectrophotometrically at 517 nm (λ_max_ for DPPH), using pure methanol as a blank. The antioxidant activity was evaluated as the percentage of DPPH radical scavenging and as Trolox equivalents (mg TE·mL^−1^). A calibration curve was prepared using Trolox (6-hydroxy-2,5,7,8-tetramethylchroman-2-carboxylic acid) as the standard antioxidant. Trolox stock solutions were prepared in 80% methanol–water (*v*/*v*) to a final concentration range of 50–200 µM, corresponding to the linear dynamic range of the assay. The calibration plot is reported in Figure S1 of the Supplementary Materials.

The DPPH scavenging activity (%) was calculated according to the following equation:

$$DPPH\;scavenging\;activity\left(\%\right)=\left[\frac{A_{0}-A_{S}}{A_{0}}\right]\times 100$$

where A_0_ = absorbance of DPPH solution alone.

A_S_ = absorbance of DPPH solution with sample.

### 2.5. Shelf-Life Determination

After the fortification procedures, fish fillets (*n* = 3 for each treatment and control group) were aseptically packaged in sterile containers, appropriately labelled, and maintained under refrigerated conditions (4 ± 1 °C) for the entire experimental period. Microbiological quality was assessed throughout storage at scheduled intervals (days 0, 3, 6, 9, 12, 15, 18, and 21) to monitor microbial dynamics and to define the microbiological shelf life according to internationally recognised standards. At each sampling time, the fillets were removed from refrigeration and allowed to equilibrate at room temperature for approximately 10 min. Subsequently, 25 g of muscle tissue were aseptically collected from each fillet and transferred into sterile stomacher bags containing 225 mL of sterile physiological saline solution (0.85% NaCl). Samples were homogenised for 2 min using a laboratory blender (Stomacher 400 Circulator, Seward Ltd., Worthing, UK). From each homogenate, serial ten-fold dilutions were prepared using sterile physiological saline, and 1 mL aliquots from suitable dilutions were plated in triplicate on selective or non-selective agar media, depending on the target microbial group. The spread-plate or pour-plate method was applied as appropriate. The microbiological analyses were carried out using several standardised methods. The total viable count (TVC) was determined on Plate Count Agar (PCA, Oxoid) and incubated at 30 °C for 72 h in accordance with ISO 4833-1:2013 [21]. *Enterobacteriaceae* were assessed on Violet Red Bile Glucose Agar (VRBG, Oxoid), with plates incubated at 48 °C for 24 h following ISO 21528-2:2017 [22]. *Pseudomonas* spp. were enumerated on Pseudomonas Selective Cetrimide Agar, incubated at 37 °C for 24 h according to ISO 13720:2010 [23]. Yeasts and moulds were analysed on Malt Extract Agar (MEA), with incubation at 30 °C for five days following the FDA BAM method [24].

Microbial counts were expressed as colony-forming units per gram of sample (CFU/g). The dilutions allowed a direct count of colony-forming units per mL (CFU/mL) on the Petri dishes, the value of which was converted to CFU/g of product. The microbiological shelf life was defined as the storage time after which at least one microbial parameter exceeded the established acceptability limits or when the presence of pathogenic species was detected.

### 2.6. Colorimetric Analysis

Colour measurements were performed on both raw and cooked fillets from each experimental group (TRT-VI, TRT-SCE, CTR). The instrumental colour analysis was carried out using a portable colourimeter (CR-400 Chroma Meter, Konica Minolta, Tokyo, Japan) with a pulsed xenon arc lamp average daylight illuminate D65, a standard observer at 10°, and The CIE L*a*b* colour scale was exploited to measure the parameters L* (lightness), a* (red-green component), and b* (yellow-blue component) that were recorded at three different points on the surface of each fillet to minimise spatial variability. For cooked samples, measurements were taken immediately after grilling in a domestic grill (200 °C, 4 min per side, core temperature ~60 °C) and before serving, while for raw samples readings were conducted after thawing and equilibration at room temperature (20 °C). Each measurement was performed in triplicate.

### 2.7. Sensory Analysis

The panel was composed by 15 assessors (gender, 8 female, 7 male; age range, 25–55 years, average 37 yrs) with over 120 h of training in descriptive analysis of seafood samples. Frozen fish fillets, both fortified and unfortified, were preliminarily thawed overnight at 4 °C and then steam-cooked at 100 °C for 10 min. The weight of each fillet was about 120 g, and the thickness was about 15 mm. Neither dressings nor salt were added to the fish during and after cooking. Fish was served warm in foam bowls and was consumed within individual sensory booths under controlled temperature and light. Between tests, reverse osmosis filtered water and unsalted crackers were assumed by assessors to rinse the palate and to reset taste buds. Samples were divided into two groups, control and treated, the latter meaning the TRT-SCE group. Samples were randomly and blindly administered to assessors (one CTR and one TRT for everyone). Panellists were invited to express their evaluation on a 9-point scale, going from 1 = extremely disagree/dislike to 9 = extremely agree/like. Attributes were categorised as follows: odour (artichoke, butter, seafood, sardine); appearance (colour intensity, colour uniformity, brightness); texture (chewiness, tenderness, firmness, fibrous, moisture); taste (astringency, salty, fat, fish oil, bitterness, aftertaste); and finally, overall acceptance.

### 2.8. Extraction of Phenolic Compounds

#### 2.8.1. Artichoke Powder

The pretreated artichoke waste was added with a solution made of ethanol and water (50:50, *v*/*v*). The suspension was stirred for three hours at ambient temperature and finally filtrated. The filtrate was subjected to this procedure thrice; eventually, the three resulting solutions were combined and transferred to the rotating evaporator to obtain a dry extract. Afterward, the extract was defatted by means of n-hexane addition, followed by sonication. Again, after paper filtration, the solution was brought to dryness and the dry extract redissolved in ethyl acetate. After filtration and evaporation, the dry extract was ready for HPLC analysis. Prior to injection, the artichoke dry extract was dissolved in methanol (50 mg/mL). An aliquot of 20 μL was injected into the HPLC system.

#### 2.8.2. Fish Fillets

Fish fillets were preliminarily thawed and homogenised in a mortar. The excess water was removed through vacuum filtration. Afterward, about 5 g of homogenised fish tissue was added to 15 mL of a methanol/water (80:20, *v*/*v*) solution. After 2 min of centrifugation, the mixture was sonicated for 15 min at 25 °C. Again, after centrifugation, the upper methanolic phase was collected. This procedure was repeated another time and the two extracts were combined. This resulting solution was filtrated through 0.22 μm nylon filters. An aliquot of 20 μL was injected into the HPLC system.

### 2.9. HPLC Analysis

Both artichoke and treated (TRT-VI and TRT-SCE) fish samples underwent HPLC analysis for the elucidation and quantification of the polyphenolic fraction. Prior to injection, samples (975 μL) were spiked with a fixed amount of syringic acid (25 μL of a 50 ppm solution) as an internal standard and filtered through syringe filters. Analyses were carried out on a Shimadzu Prominence LC-20A system (Shimadzu, Kyoto, Japan) equipped with an LC-20AD feeding solvent system, DGU-20A3R degasser, CTO-20A column oven, and SPD-M20A diode array detector. The separation of analytes took place on an Ascentis C18 column, 25 cm × 4.6 mm × 5 μm, provided by Supelco-Merck (Darmstadt, Germany). The binary solvent system was composed of water/formic acid, 99.7:0.3 *v*/*v* (Solvent A); acetonitrile/formic acid, 99.7:0.3 *v*/*v* (Solvent B). The gradient elution programme was as follows: 0 min, 2% B; 7 min, 7% B; 60 min, 60% B; 75 min, 100% B. The flow rate was 1 mL/min, and the injection volume was 1000 μL. PDA acquisition was set in the wavelength range of 200–500 nm and chromatograms were extracted at 325 and 360 nm. Data were handled by means of *LabSolution* software. Peak assignment was carried out by comparing retention times and UV spectra of the following reference standards, all provided by Merck: 1,3-dicaffeoylquinic acid; 4,5-dicaffeoylquinic acid; 1,5-dicaffeoylquinic acid; 3,5-dicaffeoylquinic acid; 1-caffeoylquinic acid; chlorogenic acid; and apigenin-7-O-glucoside. Quantification of analytes was carried out through the construction of 6-point calibration curves for the following standards: apigenin-7-O-glucoside (for flavonoids), chlorogenic acid (for monocaffeoylquinic acids), and cynarin (for dicaffeoylquinic acids). For the quantification of phenolic compounds, these chemical compounds were selected because each of them is representative of the classes of phenolic substances characteristically present in artichokes. The linearity of chlorogenic acid was tested over a range of concentration from 1.0 to 1000.0 mg·L^−1^, whereas the concentration range for both apigenin-7-O-glucoside and cynarin was 0.1–160.0 mg·L^−1^. Standard compound solutions were each analysed for three consecutive times under the same analytical conditions after method optimisation.

### 2.10. Statistical Analysis

All dataset was expressed as mean ± standard deviation. The normality of the distributions and the homogeneity of variances were verified using the Shapiro–Wilk test and Levene’s test, respectively. Differences among the experimental groups (CTR, TRT-VI, TRT-SCE) for DPPH values, colorimetric parameters (L*, a*, b*), phenolic compounds, and sensory results were assessed using one-way ANOVA, followed by Tukey’s Honestly Significant Difference (HSD) post hoc test (*p* < 0.05). The effect of cooking on colorimetric variables was evaluated within each group using a paired-sample t-test. All statistical analyses were performed using XLStat (Addinsoft) integrated into Microsoft Excel for Microsoft 365/version 2025 (Microsoft Corporation, Redmond, WA, USA).

## 3. Results and Discussion

### 3.1. Antioxidant Power

The antioxidant activity of all groups was evaluated using the DPPH assay. The results, summarised in Table 1, are expressed both as the percentage of DPPH radical scavenging and as Trolox equivalents (mg TE·mL^−1^). The results highlighted a clear difference between the CTR group and the two fortified groups. Nevertheless, it should be emphasised that untreated fillets showed an intrinsic antioxidant potential (1.23 ± 0.15 mg TE·mL^−1^), which could be attributed to the presence of bioactive peptides and to polyphenolic compounds assimilated through aquaculture feeding strategies [25,26]. Fortification by vacuum immersion (TRT-VI group) resulted in a significant increase in antioxidant activity, with mean values exceeding 40% DPPH scavenging. This improvement can be explained by the fact that vacuum conditions promote the absorption and distribution of the extract within the fillets. Indeed, the pressure gradient, induced during vacuum processing, removes interstitial liquids and reduces interfacial resistance, facilitating solution uptake via the hydrodynamic mechanism of pores [27]. This finding is consistent with the literature reporting similar fortification approaches using plant-derived extracts. For example, Zhao et al. (2019) employed vacuum impregnation to incorporate fish gelatine and grape seed extract into tilapia fillets, showing a positive correlation between the technique and antioxidant activity, although the latter was assessed through biochemical markers rather than the DPPH assay [28]. Similarly, the TRT-SCE group, treated with spray coating followed by electroporation, also showed significantly higher values compared to the control, with approximately 39% DPPH scavenging. Although slightly lower than the TRT-VI groups, these values confirm that electroporation enhanced the permeation of antioxidant compounds from the extract into the fish tissue. The application of high-intensity, short-duration electric pulses transiently increase cell membrane permeability, thereby reducing diffusional resistance and facilitating solute transfer [29]. Direct comparisons with the literature on the positive correlation between electroporation and antioxidant capacity are not currently available, as this remains a relatively unexplored area. However, recent studies, such as that of Cropotova et al. (2021), demonstrated that pulsed electric field-assisted processing is highly effective in enhancing brine uptake in seabass fillets, thereby improving fish product preservation [30].

### 3.2. Shelf Life

**Figure 1:**
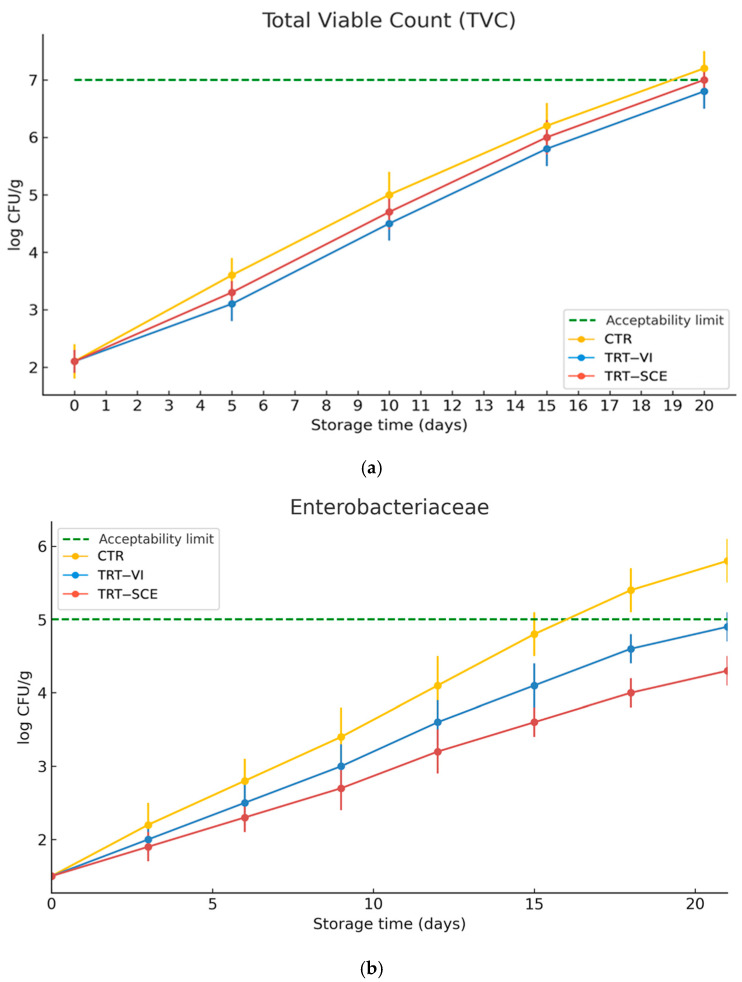

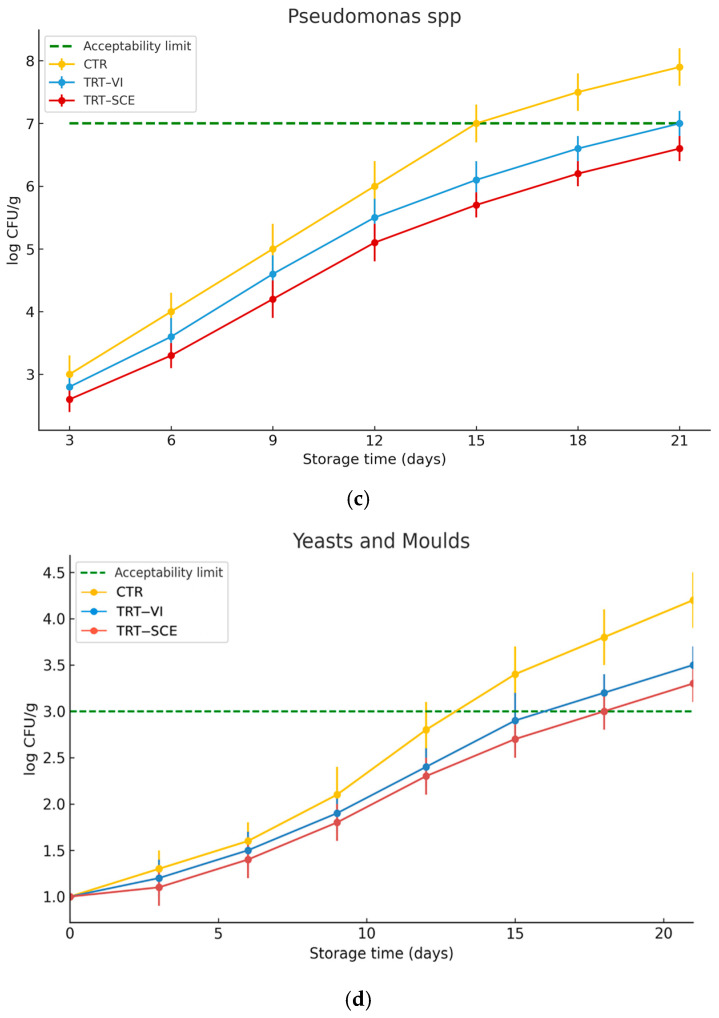
Microbiological shelf-life evaluation of raw sea bream fillets during refrigerated storage. (**a**) Total viable count (TVC), (**b**) *Enterobacteriaceae*, (**c**) *Pseudomonas* spp., and (**d**) yeasts and moulds.

Shelf-life determination was based on the growth kinetics of key spoilage microorganisms during refrigerated storage of raw sea bream fillets. Figure 1a–d reports the evolution of total viable count (TVC), *Enterobacteriaceae*, *Pseudomonas* spp., and yeasts and moulds for CTR (yellow), TRT-VI (blue), and TRT-SCE (red), with acceptability limits indicated by the green dashed line, the acceptability limits, although not strictly defined by Regulation Reg. 2073/2005, have been set by the guidelines reported by ICMSF (2002) and EFSA (2020) for each parameter analysed [31,32,33,34].

TVC increased steadily, showing a short adaptation phase (0–3 days) followed by an exponential growth. In particular, CTR exhibited the highest rate (≈0.26 log CFU g^−1^ day^−1^), exceeding 7 log CFU g^−1^ by day 19. Both treatments significantly reduced proliferation (*p* < 0.05), with TRT-VI and TRT-SCE growing at ≈0.24 and ≈0.25 log CFU g^−1^ day^−1^, respectively, and remaining below the limit throughout storage. TRT-VI maintained acceptable TVC until day 18, extending shelf life by 4–5 days, while TRT-SCE delayed the limit until day 17 [31,32,33].

Similarly, *Enterobacteriaceae* showed a slow growth until the third day, followed by rapid proliferation. CTR grew at ≈0.22 log CFU g^−1^ day^−1^ and surpassed 5 log CFU g^−1^ on day 17. Growth was significantly reduced in the fortified fillets (*p* < 0.05), with TRT-VI and TRT-SCE remaining below the limit until days 20 and 18, respectively [31,32,33,34].

*Pseudomonas spp.* exhibited a marked increase after day 3, with CTR showing the fastest rise (≈0.26 log CFU g^−1^ day^−1^) and exceeding 7 log CFU g^−1^ by day 15. In both treated groups, proliferation was noticeably slowed, postponing the limit to day 20 in TRT-VI and day 18 in TRT-SCE. The reduced bacterial growth across TVC, Enterobacteriaceae and *Pseudomonas spp.* aligns with the known antimicrobial mechanisms of artichoke polyphenols, including membrane disruption and interference with oxidative metabolism [35,36], accounting for the consistent delay in microbial development.

Yeasts and moulds showed a distinct pattern, with a longer adaptation period (0–6 days) and slower early growth, likely due to their lower initial load and reduced performance under chilled, oxygen-limited conditions. CTR exceeded the 3 log CFU g^−1^ limit at day 14, reaching 4.2 log CFU g^−1^ by day 21 [31,32,33,34]. Both treatments significantly restrained fungal proliferation (*p* < 0.05), keeping counts below the limit until days 18 (TRT-VI) and 17 (TRT-SCE), consistent with the antifungal action of artichoke polyphenols through membrane disruption, inhibition of ergosterol biosynthesis, and modulation of oxidative metabolism [35,37].

Overall, the consistent reduction in bacterial and fungal growth rates demonstrates that artichoke polyphenol fortification, most effectively via vacuum-immersion, delays spoilage processes and extends the microbiological shelf life of refrigerated sea bream fillets by approximately 4–5 days compared with the control. The greater activity of TRT-VI against TCV could be explained by the heterogeneity of the microbial populations counted with this medium. The greater activity of TRT-SCE against pathogenic microorganisms may be explained by the fact that these microorganisms are found in greater numbers on the product surface, where this type of treatment appears to be more effective.

### 3.3. Colorimetry

The colorimetric analyses were performed on the three experimental groups (CTR, TRT/VI, and TRT/SCE), both raw and cooked. The results are presented in Table 2, and they are expressed as mean ± standard deviation for the three colorimetric parameters: L* (lightness), a* (red–green component), and b* (yellow–blue component). Statistical differences within and between groups were assessed using one-way ANOVA, followed by Tukey’s HSD post hoc test.

#### 3.3.1. Raw Samples

In the control samples (CTR), L* values exceeded 58 and a* values were slightly negative, reflecting the characteristic light appearance and low myoglobin content of raw gilthead seabream fillets [37]. Both artichoke extract treatments significantly modified this chromatic profile. Vacuum immersion (TRT-VI) produced the most pronounced effect, with L* decreasing to just below 52 (≈12% reduction in surface reflectance) and marked increases in a* (~1.5) and b* (~12), indicative of surface browning and intensified yellow tones. These changes are consistent with deeper diffusion of phenolic chromophores into the muscle matrix facilitated by the vacuum re-pressurisation process, which enhances internal compound redistribution [38]. The spray coating combined with electroporation (TRT-SCE) induced milder alterations, as shown by intermediate L* (54.5), a* (~0.8), and b* (~10) values, suggesting more superficial extract penetration. One-way ANOVA followed by Tukey’s HSD test confirmed a significant treatment effect for a* and b* (*p* < 0.001), with TRT-VI differing from both CTR and TRT-SCE, and TRT-SCE remaining significantly distinct from CTR. These findings indicate that vacuum immersion promotes stronger phenolic–protein interactions, leading to modified light absorption and reflection within the visible spectrum. Increased yellow–brown pigmentation is plausibly associated with oxidizable phenolic chromophores embedded within the muscle tissue [39].

#### 3.3.2. Cooked Samples

Cooking induced substantial colour modifications across all groups. In the control fillets (CTR), L* decreased from 58.6 to 48.2, accompanied by increases in a* (−0.3 to 1.2) and b* (10.4), consistent with moisture loss, protein denaturation, surface contraction, and typical thermal pigment transformations reported for cooked fish fillets [40]. The TRT-VI group exhibited the most intense changes following heating. L* dropped to 38.9, the lowest among all treatments, while a* and b* increased to 3.6 and 16.3, respectively. These results indicate pronounced red-bronze and yellow-brown hues and support the hypothesis that vacuum immersion facilitated deeper incorporation of phenolic compounds, which likely underwent heat-induced oxidation and polymerization, thereby intensifying browning and reducing surface reflectance [38]. Chromatic shifts in TRT-SCE were intermediate: L* fell to 42.7, with corresponding increases in a* (2.5) and b* (13.9). This pattern suggests significant, yet less extensive browning compared with TRT-VI, likely due to the more superficial deposition of phenolics after spray coating and electroporation. One-way ANOVA followed by Tukey’s HSD revealed highly significant differences among cooked groups for all parameters (*p* < 0.001). TRT-VI differed significantly from both CTR and TRT-SCE across all indices, whereas TRT-SCE also differed from CTR but to a lesser extent, indicating that the fortification method modulates the extent of browning and pigment development. Within-group comparisons (raw vs. cooked) using paired t-tests (assumptions met by Shapiro–Wilk and Levene tests) showed significant changes in all parameters for all treatments (*p* < 0.001). The sharpest decrease in L* and largest increases in a* and b* occurred in TRT-VI, supporting a synergistic effect between cooking and deeply integrated phenolics, which enhanced heat-driven chromophore formation [38,40].

### 3.4. Sensory Profiles

**Figure 2:**
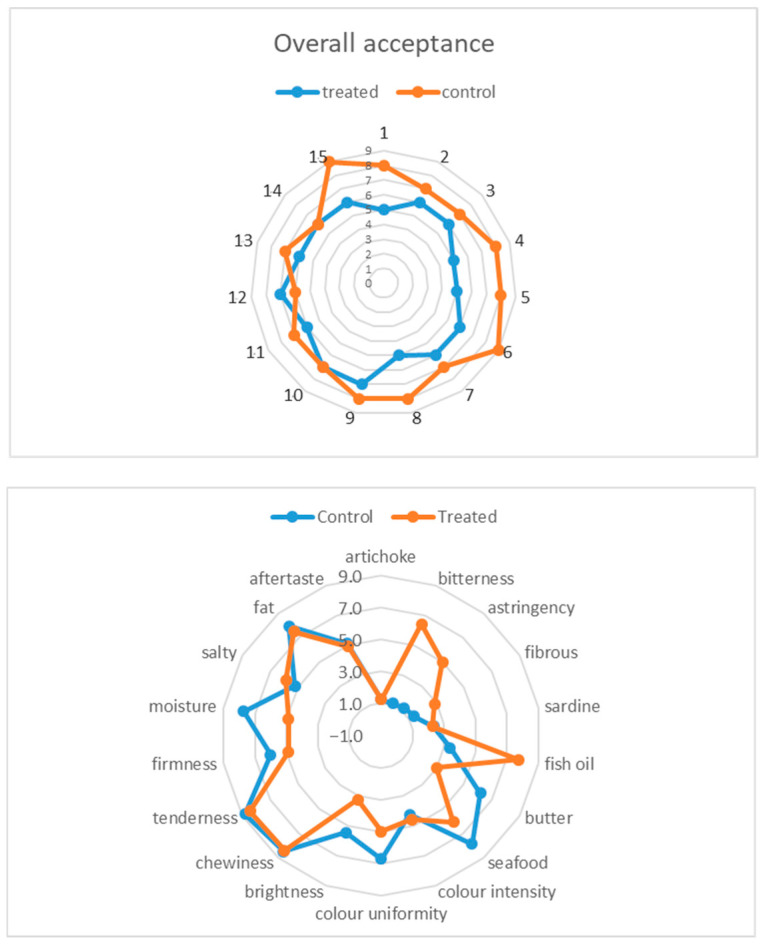
Results of sensory analysis from a panel of 15 assessors (score range, 1–9). Top: overall acceptance related to the two groups consisting of fortified (treated) and unfortified (control) fish fillets. Bottom: attributes evaluated by the panel.

Figure 2 shows the results of sensory analysis carried out on fish samples from the two groups, TRT-SCE and CTR. In view of costs, human resources, and time saving, it was decided to carry out the sensory analysis only on the TRT-SCE group of samples which had shown higher phenolic contents. In general, the overall acceptance was similar for the two types of samples, except for some specific attributes. In fact, although the control samples received slightly higher scores (~ 7.6) compared to the treated ones (~ 6.1), treated fillets still achieved values within the range of moderate to good acceptability. It can be speculated that treated samples displayed a more complex flavour profile, due to the artichoke extract. Furthermore, the treatment seems not to have compromised key textural properties and the overall sensory balance, which remained acceptable.

In particular, the highest differences between fortified and unfortified fish were recorded against the attribute’s “bitterness” and “astringency”: these two characteristics were more perceived in the TRT group of samples rather than in the CTR group (6.4 vs. 1.1; and 5.0 vs. 1.3, respectively). This finding is totally consistent with the addition in treated samples of artichoke extracts, which are notoriously characterised by such organoleptic features because of the phenolic presence [41].

Similarly, descriptors such as “fish oil” and “salty” were perceived more intensely in treated fillets, suggesting a possible enhancement of marine-related flavours due to the extract. “Butter”, “seafood”, “brightness”, and “moisture” notes received lower scores in TRT samples, indicating some sort of reduction in perceived freshness and succulence. Regarding the textural parameters such as “chewiness” and “tenderness”, these were only marginally affected, so no substantial modification has occurred in the physical structure of the fillets. From the results drawn by sensory analysis, it can be concluded that the addition of artichoke extract altered some parameters, but in general, the key elements of greatest interest to consumers remained constant. However, to overcome the slight perception of “astringent” and “bitter”, a possible strategy could be a combination of polyphenol dose reduction (tuning the amount of AE added during fortification) and the use of safe masking agents (i.e., a mild mixture of spices or protein hydrolysates).

### 3.5. Phenolic Content

The chromatographic separation was validated through the assessment of the parameters reported in Table 3 The partial least squares regression model showed good values of linearity that were close to the unit (R^2^ > 0.99). The repeatability observed by considering three replicates for each injection point was expressed as the RSD percentage (6.5% on average), demonstrating good precision as well. Because of the lack of a certified reference material, a surrogate was employed for the determination of “recovery”.

In particular, an artichoke powder sample was spiked with known amounts of the three standards and analysed separately from an unspiked artichoke sample. The HPLC results from the two analyses were compared, and the quantitative difference between spiked and neat samples corresponded to the added amount, quantified through the calibration plot. Acceptable recoveries were obtained that ranged from 86% to 95%. Limits of detection (LOD) and of quantification (LOQ), namely the lowest amount of analyte that (i) provides a response (signal) significantly different from a blank and (ii) can be measured with acceptable precision and accuracy, were measured as follows. LOD was measured according to Equation (1), while LOQ was based on Equation (2).

LOD = y_blank_ + 3*s* (1)

LOQ = 10*s*/*m* (2)

where y_blank_ is the average response of seven different blank runs; *s* is the standard deviation of the results obtained from seven very diluted sample runs; and *m* is the slope from the calibration curve. The values measured and reported in Table 2 demonstrated the LC method to be sensitive and accurate for the determination of phenolic compounds.

The phenolic compounds determined in artichoke and fish samples were caffeoylquinic acids—derivatives of hydroxycinnamic acids—and a flavonoid (apigenin glucoside), respectively. Considering all samples, their content ranged from 0.5 to 304.5 mg·100 g^−1^ (see Table 4). As expected, higher amounts of phenolics were found in artichoke powder than in fish extracts, due to the dilution effect occurring after the fortification process of fish. The opposite trend was observed only in the case of cynarin that was found to be higher in fish than in artichoke, presumably due to isomerization phenomena. The polyphenolic profile of artichoke powder agreed with previous research [42,43]. The most abundant constituents in both fish and artichoke samples were chlorogenic, 1,5-dicaffeoylquinic and 3,5-dicaffeoylquinic acids. The varied presence of polyphenols in artichoke extracts certainly affects their antioxidant response. For instance, Garbetta et al. studied the antioxidant activity of artichoke heads, concluding that the complex of polyphenols was the main contributor to this effect; they also evidenced an appreciable stability of caffeoylquinic acids in the digestive environment [44]. This aspect is strictly linked to their bioavailability, hence, to their benefits on health. Chlorogenic acid was the most abundant polyphenol in accordance with Jiménez-Moreno et al. (2019), where external bracts, leaves, and stems from artichoke waste were analysed [45]. Comparable amounts of cynarin and apigenin-7-O-glucoside were detected. In this previous work it has been emphasised how the solvent used for extraction can greatly affect the phenolic composition—i.e., water has no extraction power toward apigenin-7-O-glucoside and some hydroxycinnamic acids. If questioning the lack of caffeic acid among the phenolics listed in Table 4, it seems reasonable for this molecule to be formed during artichoke processing, due to hydrolysis [46]. It is also interesting to note the conspicuous content of chlorogenic acid, a breakdown product of hydroxycinnamic acids, forming during storage and responsible for artichoke browning [47].

As can be seen in Table 4 the polyphenolic profiles of TRT-VI and TRT-SCE groups were similar, with slightly higher content in this last group of samples.

Both treatments, namely vacuum immersion and spray coating followed by electroporation, resulted in the transfer of several artichoke-derived phenolic compounds into the fish matrix, although with different efficiencies. The TRT-SCE group of samples resulted in higher phenolic content than TRT-VI, indicating enhanced penetration when electroporation was applied. For instance, chlorogenic acid, the most abundant phenolic compound in artichoke, was significantly higher in TRT-SCE (178 mg/100 g FW) compared to TRT-VI (146.3 mg/100 g FW). To the best of our knowledge, no previous works have reported on the polyphenolic composition of fortified fish. For instance, fish fillets were added with algal extracts and their sensorial properties were investigated, whereas the polyphenolic composition was assessed only in the seaweeds [18]. Once again, algae-fortified fish burgers were prepared and evaluated on their texture and sensorial properties [48]. Amazonian freshwater fish were processed into burgers with sacha inchi oil and later investigated for their proximate composition and sensory attributes [19]; no remarks have been given on the polyphenolic profile. Conversely, in our present work, we preliminarily characterised the polyphenolic profile of the artichoke waste used as supplementation material in fish; afterward, polyphenols were quantified in fortified fish as well.

## 4. Conclusions

This study demonstrates that the fortification of sea bream (*Sparus aurata*) fillets with artichoke-derived polyphenols is an effective and technologically feasible strategy to enhance microbiological stability, antioxidant capacity, and overall functional value while preserving acceptable sensory quality. Both post-harvest biofortification approaches investigated, vacuum immersion (TRT-VI) and spray coating followed by electroporation (TRT-SCE), successfully inhibited the growth of spoilage microorganisms. Among them, TRT-VI consistently exerted the strongest antimicrobial effect, resulting in a shelf life extension of approximately 4–5 days compared with untreated controls.

Phenolic profiling revealed that fortified fillets contained considerable amounts of bioactive compounds, mainly caffeoylquinic acids such as chlorogenic, 1,5-dicaffeoylquinic, and 3,5-dicaffeoylquinic acids. The incorporation of these compounds into the fish matrix directly contributed to the enhanced antioxidant capacity observed in treated samples, supporting the potential of polyphenol-enriched fish products as value-added functional foods. Sensory evaluation further demonstrated that, despite moderate increases in bitterness and astringency, fortified fillets maintained good overall acceptability, with no significant alterations in texture, indicating that biofortification can be achieved without compromising key consumer-relevant attributes.

Based on the analysis of compositional, functional, and processing data, the two fortified samples exhibited complementary advantages. TRT-SCE achieved slightly higher polyphenol incorporation, suggesting greater nutritional enrichment at the compositional level, whereas TRT-VI demonstrated superior functional performance, as reflected by the higher antioxidant activity and a more pronounced extension of microbiological shelf life. From a production perspective, both treatments relied on the same low-cost raw materials and identical extraction procedure; however, the spray-coating-based approach may be considered more cost-effective and efficient at the industrial level, owing to faster processing, easier integration into existing processing lines, and reduced handling of liquid extract compared with vacuum immersion.

From an applied and industrial standpoint, these findings provide a solid basis for the transfer of this biofortification strategy to seafood processing chains. The use of food-grade, plant-derived extracts recovered from agro-industrial by-products, in combination with scalable post-harvest technologies, such as vacuum immersion and surface treatments, aligns with current industry demands for natural preservation solutions, clean-label formulations, and sustainability-driven innovation. Overall, this work supports the realistic integration of botanical antioxidants into aquaculture-derived fish products, offering a practical pathway for the development of value-added functional seafood that meets both market expectations and public health objectives. Future research should focus on process optimisation at pilot and industrial scales, comprehensive cost-effectiveness assessments, validation of product performance under commercial storage and distribution conditions, and confirmation of health-related benefits through in vivo and clinical studies.

## Figures and Tables

**Table 1:** Antioxidant activity of control (CTR) and fortified fillets (TRT-VI and TRT-SCE) expressed as percentage of DPPH radical scavenging and Trolox equivalents (mg TE/mL). Values are presented as mean ± standard deviation.

Sample Group (*n* = 20)	DPPH Scavenging Activity (%)	Antioxidant Capacity (mg TE·mL^−1^)
CTR	19.6 ± 1.5	1.23 ± 0.15
TRT-VI	42.5 ± 3.2	3.56 ± 0.22
TRT-SCE	38.9 ± 2.8	3.06 ± 0.18

**Table 2:** Mean values ± standard deviation of colorimetric parameters in raw fish fillets, with statistical significance evaluated via ANOVA and Tukey HSD tests.

Group	Treatment	L* (Lightness)	a* (Red Green)	b* (Yellow Blue)
**CTR**	Raw	58.6 ± 1.3 ^a^	−0.3 ± 0.4 ^a^	7.5 ± 0.9 ^a^
	Cooked	48.2 ± 1.5 ^a^	1.2 ± 0.3 ^a^	10.4 ± 0.8 ^a^
**TRT-SCE**	Raw	54.5 ± 1.4 ^b^	0.8 ± 0.5 ^b^	10.2 ± 1.1 ^b^
	Cooked	42.7 ± 1.6 ^b^	2.5 ± 0.4 ^b^	13.9 ± 1.0 ^b^
**TRT-VI**	Raw	51.7 ± 1.2 ^c^	1.5 ± 0.6 ^c^	12.0 ± 1.2 ^c^
	Cooked	38.9 ± 1.3 ^c^	3.6 ± 0.5 ^c^	16.3 ± 1.1 ^c^

Superscript letters (a, b, c) indicate statistically significant differences between groups within each column, based on one-way ANOVA followed by Tukey’s HSD post hoc test (*p* < 0.05). Groups not sharing the same letter in the same column are significantly different.

**Table 3:** Validation parameters of the HPLC method used for phenolic compounds separation and quantification.

Analyte	Linearity (R^2^)	Repeatability (RSD%)	LoQ (mg·Kg^−1^)	LoD (mg·Kg^−1^)	Recovery (%)
Chlorogenic acid	0.998	5.2	0.025	0.005	95
Apigenin-7-O-glucoside	0.994	7.5	0.046	0.013	86
Cynarin	0.997	6.8	0.044	0.037	93

**Table 4:** Phenolic compounds determined in artichoke and fish samples.

Compound	Artichoke (mg/100 g, FW)	Fish TRT-VI (mg/100 g, FW)	Fish TRT-SCE (mg/100 g, FW)
1,3-dicaffeoylquinic acid (cynarin)	4.3 ± 0.8	5.6 ± 0.7	6.2 ± 0.9
4,5-dicaffeoylquinic acid	23.2 ± 2.2	9.8 ± 0.9	11.2 ± 1.1
1,5-dicaffeoylquinic acid	185.7 ± 11.4	55.4 ± 2.8	59.4 ± 3.1
3,5-dicaffeoylquinic acid	158.4 ± 9.7	38.3 ± 3.4	45.2 ± 2.5
1-caffeoylquinic acid	31.3 ± 1.3	<0.1 ± 0.0	1.1 ± 0.4
chlorogenic acid	304.5 ± 12.7	146.3 ± 13.6	178 ± 9.4
apigenin-7-O-glucoside	15.8 ± 1.2	<0.1 ± 0.0	1.4 ± 0.4

## Data Availability

The original contributions presented in the study are included in the article/Supplementary Material, further inquiries can be directed to the corresponding author.

## Supplementary Materials

The following supporting information can be downloaded at: https://www.mdpi.com/article/10.3390/foods15010175/s1. Figure S1: Trolox calibration curve used in DPPH antioxidant assay. Figure S2: External standard calibration curve of cynarin. Each point is the result of triplicate injections. Figure S3: External standard calibration curve of apigenin-7-O-glucoside. Each point is the result of triplicate injections. Figure S4: External standard calibration curve of chlorogenic acid. Each point is the result of triplicate injections.

## Abbreviations

The following abbreviations are used in this manuscript:

PUFAs	Polyunsaturated fatty acids
DHA	Docosahexaenoic acid
EPA	Eicosapentaenoic acid
VI	Vacuum immersion
SCE	Spray coating and electroporation
AE	Artichoke extract
CTR	Control
TRT	Treated
DPPH	2,2-Diphenyl-1-picrylhydrazyl
CFU	Colony forming unit
HSD	Honestly significant difference
TVC	Total viable count
LOD	Limit of detection
LOQ	Limit of quantification
