# Supplementary material for: Biofortification of Sea Bream Fillets with Artichoke Polyphenols: Effects on Antioxidant Capacity, Shelf Life, and Sensory Quality
Source: Foods. 2026 Jan 5;15(1):175. doi: 10.3390/foods15010175 (PMC12785955; doi:10.3390/foods15010175)
Supplement: Supplementary file 1 [file foods-15-00175-s001.zip › foods-4048818-supplementary.pdf]

Supplementary material

Biofortification of fish fillets with artichoke by-product extracts: impact on nutritional value, antioxidant capacity, and shelf life

Rossella Vadalà [1,†], Giovanna Lo Vecchio [1,†], Laura De Maria [1], Daniela Metro [1], Roberta Tardugno [2], Nicola Cicero [1,3,4,*] and Rosaria Costa [1]

[1] Department of Biomedical, Dental, Morphological, and Functional Image Sciences (BIOMORF), University of Messina, Via Consolare Valeria 1, 98125 Messina, Italy; rvadala@unime.it (R.V.); giovanna.lovecchio@unime.it (G.L.V.); laura.demaria@unime.it (L.D.M.); daniela.metro@unime.it (D.M.); costar@unime.it (R.C.)
[2] Department of Pharmacy-Drug Sciences, University of Bari 'Aldo Moro', Via Orabona 4, 70125 Bari, Italy; roberta.tardugno@uniba.it
[3] Institute for Agriculture and Forestry Systems in the Mediterranean, National Research Council of Italy, Via Empedocle 58, 95128 Catania, Italy
[4] Science4life srl, University of Messina, 98168 Messina, Italy
* Correspondence: nicola.cicero@unime.it
† These authors contributed equally to this work.

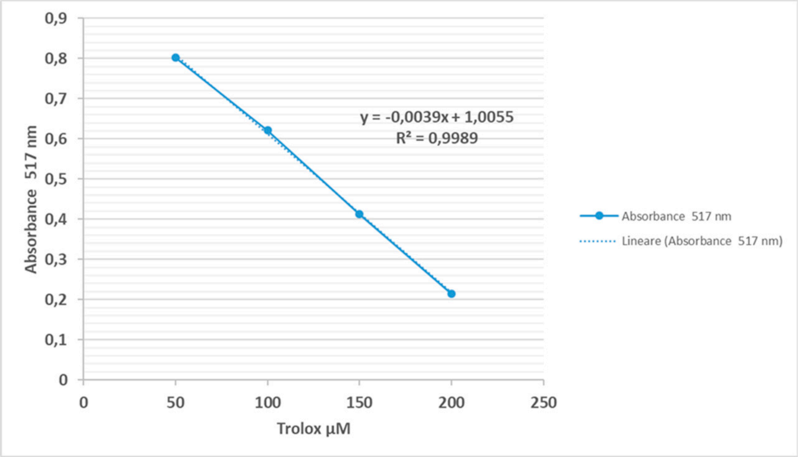

Figure S1. Trolox calibration curve used in DPPH antioxidant assay.

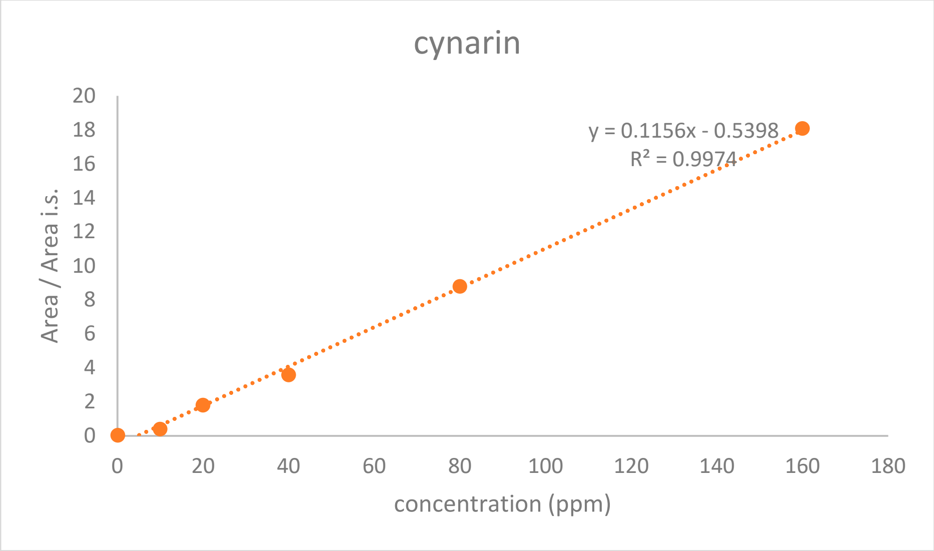

Figure S2. External standard calibration curve of cynarin. Each point is the result of triplicate injections.

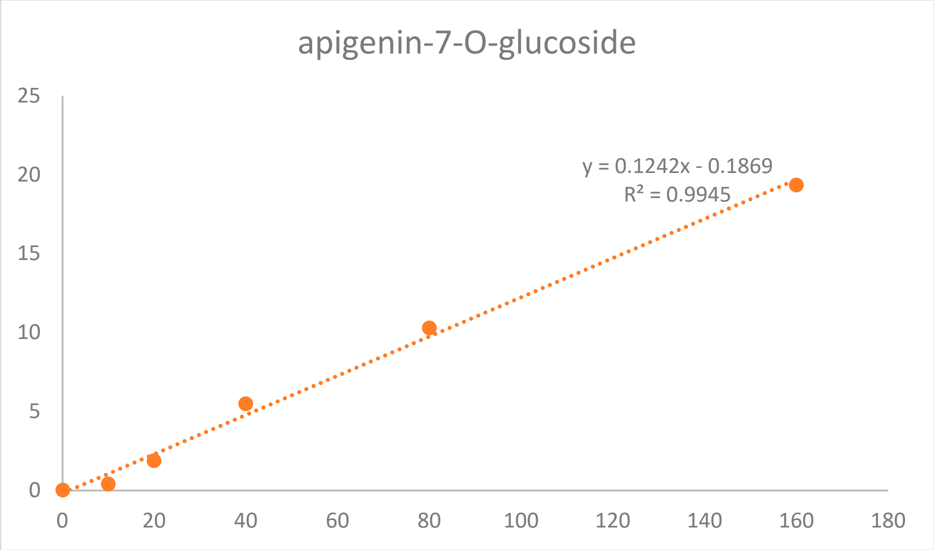

Figure S3. External standard calibration curve of apigenin-7-O-glucoside. Each point is the result of triplicate injections.

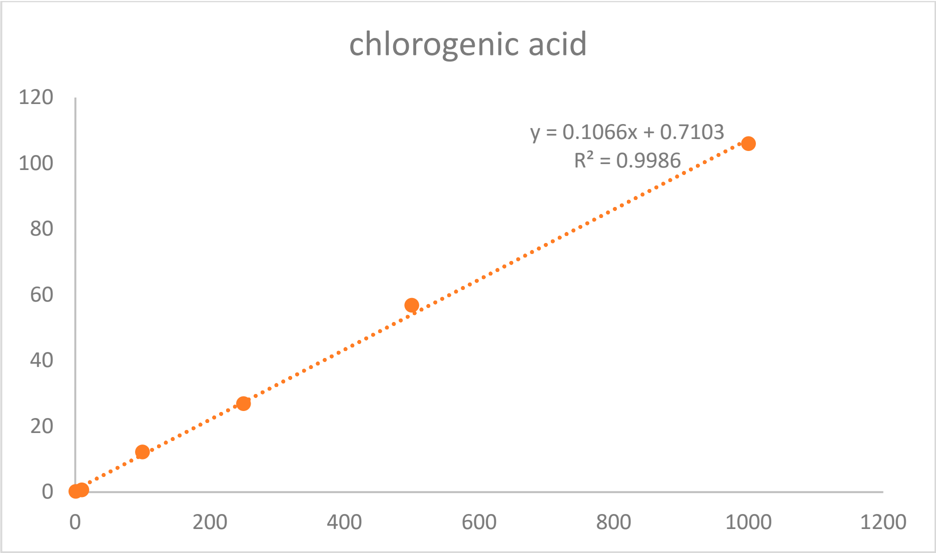

Figure S4. External standard calibration curve of chlorogenic acid. Each point is the result of triplicate injections.
